# Out-of-hospital cardiac arrest

**Published:** 2016

**Authors:** PJ Commerford

Terminology may drive our perceptions of activities and our actions in response to the events we see occurring around us. Is it time to take the P (pulmonary) out of CPR (cardiopulmonary resuscitation) when referring to witnessed out-of-hospital cardiac arrests (OHCA)? I was prompted to consider this when I was personally involved in resuscitation of an individual who suffered an out-of-hospital cardiac arrest when walking his dog, as I was walking mine, in a park near our home. He collapsed a few metres in front of me, immediately after tossing a ball for his dog to chase, and when I examined him he was wheezing, cyanosed and pulseless.

I immediately started compression-only CPR (CO-CPR) as I understood this was the accepted standard. It was gratifying to see the cyanosis resolve. Fortunately paramedics with a defibrillator arrived within 15 minutes and the patient was transferred to hospital in a stable condition and discharged, neurologically intact, after implantation of an implantable cardiac defibrillator.

I was interested in the responses of fellow citizen bystanders, some of whom felt that compression only, neglecting ventilation, was incorrect, and tried to correct my approach. First-responder ambulance personnel similarly seemed anxious to interrupt chest compression to place an oral airway despite the fact that the patient was pink (as opposed to earlier cyanosis) and there was audible air exchange.

By happy coincidence, shortly after that incident, I reviewed, for our departmental journal club, an article that reinforced my opinions and one that I believe should be more widely disseminated. In a perspective article in Circulation, Gordon Ewy1 clearly describes the benefits of CO-CPR for witnessed out-of-hospital arrest, the experimental animal work supporting it, and its successful implementation in the state of Arizona. 

The results were impressive. In all patients with OHCA, the survival rate was 7.8% in those receiving guidelines CPR and 13.3% in those receiving CO-CPR. In the subset of patients with witnessed cardiac arrest and a shockable rhythm, survival rate was 17.7% in those receiving guidelines CPR and 34% in those receiving CO-CPR.

It is emphasised that this applies to OHCA were oxygenation immediately prior to the arrest is normal, and does not apply in other circumstances, such as in hospital, where hypoxia may in fact contribute to the arrest. This report documents succinctly and clearly one of the few real advances and successes in the management of witnessed out-of-hospital cardiac arrest in several decades and should be read and widely disseminated.
